# Some War Problems in Food

**Published:** 1917-03-24

**Authors:** 


### SOME WAR PROBLEMS IN FOOD.*

### II. Meat Rations for the Staff.

THERE is no doubt that the war rations have led many people to find out that their butcher's bill has been out rageously too high. This is markedly the case in institutions. Owing to the stupidity of cooks and the incompetence of housekeepers it has been the custom in far too many households, large and small, to satisfy the healthy appetites of the staff on solid chunks of meat, hot or cold, according to the time of day. Enormous joints came and went. Such as were too deeply cut into to make a respectable appearance again were often wasted, according to various time-honoured methods of waste, beginning with the "perquisite of the cook" and ending with the pig-pail. Extravagant rationing, too, has been a very fertile source of meat wastage. Poor-Law officers are, we believe, still entitled to draw to the extent of one pound of meat per head a day, in addition to some two pounds of bacon and one fowl per week. These amounts are preposterous, and must have been the result of pure guess-work on the part of some administrator with a strong carnivorous instinct. Half a pound of uncooked meat a day per head should afford the housekeeper sufficient to supply all reasonable needs. But the problem now is to reduce this amount to 2½ lb., or 40 oz., per head a week, a matter which requires some management.

It ought to be quite frankly realised that the war ration does not admit of the appetite being satisfied on meat dishes three times a day. People who have been accustomed to consume slice after slice of cold ham or bacon for breakfast, a twice-piled plate of roast beef or mutton for their mid-day meal, and a supper consisting mainly of cold meat will have to alter their diet very considerably to conform with war conditions. It does not follow that they need be under-fed. But to avoid some apparent discomfort and discontent the housekeeper will have to use her brains.

Our canny French neighbours, with their culinary talents, discovered centuries ago the comparative extravagance of roast and baked meats. We have already pointed out the extent to which meat is reduced in bulk and weight by being subjected to dry heat. When cooked with the aid of steam or water it increases in bulk, and its digestible qualities are improved. However partial the staff may be to the traditional roast

(*Continued on p.* 506.)

* Previous articles appeared on February 24 and March 10.

### SOME WAR PROBLEMS IN FOOD—

(*Continued from page 504.*)

joint, it should be regarded for the present as a costly luxury, to be served sparingly if at all. Institution cooks must be set to master the art of preparing really savoury stews which, with an admixture of rice or other cereals, will be found both satisfying and satisfactory. Beans, macaroni, spaghetti, oatmeal, barley, and such vegetables as carrots, onions, and turnips should either form a part of such stews or be served with them, and will soon be secure of a welcome. With such aids the mid-day meal should rather gain than lose, but the reduction of the meat ration must be countered by the addition of more variety.

There is, however, the question of supper to be faced. We plead in this connection for the restoration of soup to its proper place in the institution household. The very high food value of well-made, thick soup is unduly ignored by most housekeepers in this country. Taking a lesson again from the French, it would speedily be discovered what a valuable and inexpensive form of nourishment is here ready to hand. For in every large household the bones, with the odds and ends of meat trimmed off by the cook or left over on the dishes after carving, are capable of being turned to rich account in the stock-pot. This, with the aid of cereals, should provide sufficient for the principal supper dish for the whole staff every day. Some articles of diet, like split peas, for instance, lentils, and semolina, are never really appetising unless served as a thickening in soup, and these are of the highest nutritive value. The soup course is particularly suitable for tired workers; and who is not tired at the end of a hospital day? It feeds and satisfies at the same time, and is far more than the equivalent, from the meat-eater's point of view, of a cut from the cold joint.

Breakfast we have left out of consideration, partly because there are substitutes such as fish and eggs which can replace bacon, and partly because the consumption of meat at the early hour dictated by hospital necessities is indefensible from physiological reasons. The stomach at 7 or 8 A.M. is not ready for heavy tasks, and none who have given a fair trial to a light but nourishing breakfast will surrender the extra vigour thus secured. Be it remembered also that breakfast in hospitals is invariably followed two or three hours later by a light intermediary repast.

